# Annual Spending per Patient and Quality in Hospital-Owned Versus Physician-Owned Organizations: an Observational Study

**DOI:** 10.1007/s11606-019-05312-z

**Published:** 2019-09-03

**Authors:** Vivian Ho, Leanne Metcalfe, Lan Vu, Marah Short, Robert Morrow

**Affiliations:** 1 Baker Institute for Public Policy at Rice University, Houston, TX USA; 2 Department of Economics, Rice University, MS 22, 6100 Main Street, Houston, TX 77005 USA; 3 Department of Medicine, Baylor College of Medicine, 1 Baylor Plaza, Houston, TX 77030 USA; 4 Blue Cross Blue Shield of Texas, Chicago, IL USA

**Keywords:** cost analysis, health economics, health policy, physician behavior

## Abstract

**Background:**

Recent studies that compared patient spending in hospital-owned physician practices versus physician-owned groups did not compare quality of care. Past studies had incomplete measures of physician-hospital integration, or lacked patient-level data.

**Objective:**

To measure the association between physician-hospital integration and both spending and quality using patient-level data and explicit physician-hospital contracting information.

**Design:**

Retrospective review of claims data from 2014 through 2016. Adjustments were made for patient, physician, and regional characteristics.

**Patients:**

Patients aged 19 to 64 enrolled in a Blue Cross Blue Shield Texas Preferred Provider Organization in the four largest metropolitan areas in Texas who could be attributed to a physician practice based on claims.

**Main Outcomes and Measures:**

Annual spending per patient was compared for patients treated by a physician practice that is billing through a hospital, versus billing through an independent physician practice; spending was also subdivided by BETOS category, by site and type of care, and percent of patients with positive spending by subcategory. Quality measures included readmission within 30 days of discharge for hospitalized patients, appropriate care for diabetic patients, and screening mammography for women ages 50–64.

**Results:**

Estimates suggest that patients in a preferred provider organization incur spending which is 5.8 percentage points higher when treated by doctors in hospital-owned versus physician-owned practices (95% CI 1.7 to 9.9; *p* = 0.006). Spending is significantly higher for durable medical equipment, imaging, unclassified services, and outpatient care. The spending difference appears attributable to greater service utilization rather than higher prices. There was no consistent difference in care quality for hospital-owned versus physician-owned practices.

**Conclusions and Relevance:**

We find that financial integration between physicians and hospitals raises patient spending, but not care quality. Given that higher spending raises the price of health insurance, policy makers should carefully consider policies that limit consolidation of hospitals and physicians.

**Electronic supplementary material:**

The online version of this article (10.1007/s11606-019-05312-z) contains supplementary material, which is available to authorized users.

## INTRODUCTION

In the past 15 years, hospitals have pursued tighter integrative relationships with physicians, including direct employment of doctors. In 2003, approximately 29% of hospitals had at least some employed physicians, a number that rose to 42% by 2012.^1^ The share of US physician practices owned by hospitals rose from 14% in 2012 to 29% in 2016.^2^ Economists refer to these tighter relationships between hospitals and physicians as vertical integration, because they represent the decision of hospitals to exert increasing control over physicians as an essential input to inpatient care.

Experts offer multiple reasons for why hospitals and physicians might pursue closer contractual relationships. Physician integration could lead to efficiency gains by lowering transaction costs and improving efforts to manage and coordinate care.^3^ Some experts hypothesized that vertical integration is an unintended consequence of Medicare’s Accountable Care Organization (ACO) model, which promotes care coordination.^4, 5^ However, a recent study found no association between local market ACO penetration and increased vertical integration.^6^

Vertical integration could instead result from a desire by hospitals and physicians to earn higher revenues. If a hospital controls a large share of physicians in a region, the hospital may be able to negotiate higher prices for its services from insurers, due to the facility’s stronger bargaining power.^3, 7^ Hospitals and physicians may also be taking advantage of the fact that Medicare reimburses hospital-based outpatient care at higher rates than similar care provided by independent physician practices.^3, 8^

Several previous studies have found that vertical integration is associated with higher annual spending, but none of these studies concurrently measured the relation between vertical integration and quality. Additional shortcomings faced by these studies included access to only region- or practice-level versus patient-level data.^9–11^

This study analyzes insurance claims from Blue Cross Blue Shield of Texas (BCBSTX) for 2014 through 2016 in Texas to compare annual health spending and quality of care for patients treated by doctors in hospital-owned versus physician-owned practices. Texas is the second most populous state in the nation, and BCBSTX is the largest large-group insurer in the state, with 48% market share.^12^ We analyze preferred provider organization (PPO) insurance claims, which is more common than health maintenance organizations (HMO) coverage in the USA (161.5 million PPO versus 89.3 million HMO).^13^ Unlike past studies, we directly observe vertical integration through insurance contracts rather than inferring it through survey or claims data. We also control for unmeasured factors that systemically vary across regions.

## METHODS

### Data on Healthcare Expenditures

We analyzed all preferred provider organization insurance claims processed for care through BCBSTX for 2014 through 2016 in the four largest Texas metropolitan statistical areas (MSAs; Austin, Dallas, Houston, and San Antonio). The population in these MSAs totaled 18.9 million in 2017,^14^ which is greater than the entire population of 46 other US states.^15^ The sample includes all claims for healthcare services, except for prescription of drugs.

For each patient, we summed all claims in each calendar year to calculate the annual allowed amount for each patient, which represents payments to providers by BCBSTX, as well as out-of-pocket expenses (deductibles and copayments) that the patient is responsible for. We refer to the allowed amount on each claim as the price for that particular service. Physicians influence patient spending within their practice, as well as what hospitals or other facilities that patients may be referred to outside of their practice.^16^ Thus, annual spending for each patient represents all services used by patients, not just the services directly provided by the attributed physician organization. Patients with > $100,000 in costs in a calendar year were excluded from the sample, in order to exclude the effect of small numbers of very sick patients on average expenditures per patient.^11, 17^ A flow diagram illustrating the change in sample size resulting from each exclusion is in Appendix Figure 1.^18^

In addition to comparing overall spending by physician ownership type, we decomposed spending by clinical category, as defined by the Berenson-Eggers Type of Service (BETOS) classification system, and by site and type of care. The BETOS coding system was developed by the Center for Medicare and Medicaid Services to analyze growth in Medicare expenditures, grouped by readily understood clinical categories. BETOS codes are assigned to each Health Care Procedure Coding System (HCPCS) code.^17, 19^

### Attributing Patients to Physician Organizations

We limited the analysis to adults ages 19 to 64 who were continuously enrolled in at least one of the calendar years 2014 to 2016. Ideally, we would have attributed each patient to a primary care physician (PCP) in each calendar year based upon the PCP who accounted for the majority of their visits in that same year. But if we followed this approach, patients with no visits to a PCP in a calendar year could not be attributed to a physician. Therefore, we searched for claims for visits to a PCP in a 24-month window that included the calendar year and additional months closest to that year. Additional details of our attribution method are provided in Appendix eMaterial 1.^18^ Primary care physicians were those doctors who listed their specialty as family practice, general practice, geriatrics, internal medicine, or pediatrics.

### Quality Measures

Among hospitalized patients, we identified those readmitted within 30 days of discharge. We also constructed four process measures adapted from the Healthcare Effectiveness Data and Information Set (HEDIS; National Committee for Quality Assurance); for patients with diabetes, hemoglobin A_1c_ and LDL cholesterol screening, and diabetic retinal examination; screening mammography for women ages 50–64 years.

### Defining Physician Organizations

We determined the ownership status of physicians based on their recorded network for reimbursement in the BCBSTX internal database. Each claim contains the tax identification number (TIN) of the treating physician or hospital. The Network Management group at BCBSTX maintains records of the contracts negotiated with physician and hospital organizations, which also contain information on the identities of physicians included in each contract. These groupings were used to assign each TIN to a particular physician or hospital organization.

We compared the annual healthcare spending for patients attributed to a physician treated by local hospital-owned physician groups versus multi-hospital system–owned physician groups, versus physician-owned organizations. Following past research, local-owned hospital organizations are single hospitals or hospital chains which do not extend across geographic regions. Multi-hospital system–owned physician groups are affiliated with a hospital system that extends across geographic regions.^11^

### Control Variables

The annual expenditures for each patient were adjusted for the relative disease burden of each patient, differences in the cost of delivering care across regions of Texas, the annual number of patients attributed to each physician organization, whether the patient was in a consumer-directed health plan (CDHP), and unobserved but systemic differences in spending across the four MSAs by year.

A CDHP combines a high deductible health plan (HDHP) as defined by the Internal Revenue Service (IRS) with a health savings account or a health reimbursement account. The minimum annual deductible for an HDHP was $1300 for self-only coverage and $2600 for family coverage.^20^ Patients with CDHPs may be less likely to use healthcare services than patients without these plans.

Relative disease burden was accounted for using patient age categories, gender, and a concurrent risk score. BCBSTX calculates the concurrent risk score based on claims in the calendar year of treatment using the Verscend Technologies DxCG concurrent risk score. The Verscend DxCG is a risk scoring model that performs as well as, or better than, other available risk models in predicting the current year’s healthcare expenditures based on diagnoses reported in the claims.^21^

Differences in the cost of care across regions of Texas were accounted for by including as a control variable the wage index that is used to construct the geographic adjustment factor (GAF).^22^ The GAF is used by the Center for Medicare and Medicaid Services to adjust fee-for-service reimbursement rates for regional differences in input prices and is updated yearly. The wage index reflects the average hourly hospital wage in each local market divided by the national average hourly hospital wage.^23^

### Statistical Analyses

Annual payments by physician organization type were first compared using descriptive statistics. We then estimated regression-adjusted analyses, distinguishing both local hospital-owned and multi-hospital-owned physician groups from physician-owned practices. Given that the estimated spending differences between the two hospital-owned practice types were not statistically significantly different from each other, we report regression-adjusted spending differences for physician-owned practices versus all hospital-owned organizations combined.

We estimated spending regressions using all expenditures as the dependent variable, as well as spending by BETOS category and site and type of care. The regressions were estimated using SAS Enterprise Guide, version 7.1. In these regressions, the explanatory variable of interest is an indicator for whether the patient was treated by a hospital-owned or physician-owned practice. We then decomposed the spending change into a price effect and a utilization effect by replacing the price on each claim with the median price in its respective BETOS category. Spending regressions estimated using the annual sums of standardized claims reflect only differences in utilization.^17, 24^ To further understand the source of spending differences by organization type, we compared the percent of patients with positive (versus no) annual spending in each BETOS category and site and type of care for patients treated by physician-owned versus hospital-owned practices.

The number of patients attributed to each physician group was specified in four categories: practices with up to 500 patients; 501 to 10,000 patients; 10,001 to 15,000 patients; and 15,001+ patients per year. These categories were chosen to obtain a broad separation of practice sizes within our sample, but also reflect information from previous studies on physician practice panel size and the distribution of physicians by practice size.^3, 25^

The regressions include year fixed effects to measure trends in spending over time, as well as the control variables outlined above. Patient age was included using categorical indicator variables for ages 19 to 29, 30 to 39, 40 to 54, and 55 to 64. The DxCG concurrent risk score was specified using categorical indicators for deciles of patient values in our sample, to allow for flexibility in how patient risk influences spending as patient illness severity increased. The wage index and its squared value were also included.

Interactions of indicator variables for the four MSAs multiplied by year indicator variables control for unobservable systematic factors (e.g., regional practice patterns) that may be correlated with both vertical integration and spending. With the inclusion of these variables, the regression results yield estimates of spending differences associated with practice ownership-type within MSAs and year, unconfounded by unmeasured differences across MSAs and over time.

Annual spending in dollars for each patient was converted to logarithmic units in order to obtain regression estimates expressed in percentage differences associated with each covariate. The regressions were estimated using a quasi-likelihood generalized linear model with a log link. For cases where a patient has $0 spending in a BETOS category, this approach includes these observations, whereas they would be excluded from an ordinary least squares regression with the natural log of spending as the dependent variable.^26^

Following recent studies, we also estimated multivariate regressions with spending expressed in dollar units rather than logarithmic units.^11, 24, 27^ These studies state that in large samples, linear models generally outperform other statistical models at estimating population averages, even though they can be less precise at estimating the tails of a spending distribution.^26, 28–30^ The standard errors for all regression estimates were clustered by physician organization across years to account for heteroscedasticity.^31, 32^ An estimate is considered to be statistically significant if a two-sided *t* test yields a *p* value ≤ 0.05.

Given that previous studies observe that physicians may align with hospitals in order to bill more services in the higher reimbursement outpatient setting, we examine cases where clinicians might exercise this behavior. For BETOS categories where we find a significant difference in spending by physician practice ownership type, we searched for current procedure terminology (CPT) codes (in professional claims) that could be cross-walked to revenue codes (in outpatient claims). We then compare the number of claims per enrollee between patients treated by hospital-owned versus physician-owned practices, and we calculate the percent of claims billed in the outpatient setting.

The research protocol was determined to be exempt from review by Rice University’s Institutional Review Board, because all physician and patient identifiers were removed from the dataset made available to us for analysis.

## RESULTS

### Descriptive Statistics

The patients in the sample represent 32% of all patients with a PPO contract through BCBS in Texas who filed a claim with the insurer in this year. The number of patients attributed to a physician organization changed from 610,460 in 2014 to 562,560 in 2016. There are fewer patients in the sample in 2016, when there was a decrease in available PPO products for customers purchasing individual coverage. Table 1 in the Appendix^18^ compares the characteristics of patients included in the sample versus those excluded, by year.

Table 1 presents 2015 descriptive statistics on all physician organizations in the BCBSTX claims and the patients who could be attributed to one of these organizations. The number of physician organizations that were unaffiliated with a hospital was 1869 in 2015. There were 27 local hospital-owned physician organizations in 2015 and 11 multi-hospital-owned physician groups. The distribution of physician specialties in physician- versus hospital-owned practices is provided in Appendix Table 2.^18^

**Table 1:** **Characteristics of Physician Organizations and Attributed Patients Participating in the BCBSTX Network in 2015**

Medical groups			
	Physician-owned	Local hospital-owned	Multi-hospital system-owned
Total			
No. of physician organizations (Pos)	1869	27	11
No. of patients	477,927	66,127	77,789
Patients per PO, mean	256	2449	7072
Patients per PO, minimum range	5	8	70
Patients per PO, maximum range	64,168	29,833	40,349
Severity of patient health status (DxCG risk scores)*			
	1.25 (0.19–11.99)	1.29 (0.44–3.41)	1.22 (0.47–1.81)
Allowed medical spending	4558	5078	4800
Age	43.1	43.5	42.8

*Minimum and maximum in parentheses

The mean age and risk scores for patients in physician- versus hospital-owned practices do not look noticeably different. However, patients in physician-owned practices had lower annual spending of $4558 (95% CI $4531–$4585) compared with patients treated by local hospital-owned groups ($5078, 95% CI $5000–$5155) or multi-hospital system–owned organizations ($4800, 95% CI $4730–$4870). Similar findings were noted for physician organizations and patients in 2014 and 2016 and are reported in Table 3 of the Appendix.^18^

### Adjusted Spending Differences

Table 2 reports unadjusted and adjusted differences in spending by physician practice ownership type, BETOS category, and site and type of care. Average annual spending for patients treated by hospital-owned organizations is 5.8% (95% CI 1.7 to 9.9; *p* = 0.006) higher than for physician-owned practices. When one performs this comparison assigning median prices by BETOS category to each claim, the differential for hospital-owned organizations declines somewhat to 4.9% (*p* = 0.02).

**Table 2:** Annual Expenditure Differences per Patient for Hospital- Versus Physician-Owned Organizations, 2014–2016, Overall and by BETOS Category and Site or Type of Care

	Unadjusted spending		Adjusted percentage difference	Adjusted dollar difference
	Physician-owned	Hospital-owned	Hospital- vs. physician- owned	Hospital- vs. physician-owned
Average annual spending per patient ($)	4451.80	4824.93	5.83**	280.08***
Average annual spending per patient using median prices ($)	3141.45	3309.40	4.89*	173.51***
By BETOS category ($)				
Evaluation and management	1115.31	1144.24	2.28	33.35
Procedures	922.53	986.06	3.23	18.90
Imaging	669.04	781.39	13.00***	93.07***
Test	601.80	608.43	2.12	9.19
Durable medical equipment	315.09	356.50	12.93**	35.62***
Other	321.30	326.56	7.74	2.41
Unclassified	506.71	621.75	21.38***	87.81***
By site and type of care ($)				
Professional payments	2259.69	2223.82	− 2.55	− 71.1
Outpatient facility	1487.84	1859.11	21.66***	310.36***
Inpatient facility	704.27	742.01	− 6.64	41.08*

*p < 0.05; **p < 0.01; ***p < 0.001

The estimates are adjusted for practice size, year, age, gender, concurrent risk score, participation in a consumer-directed health plan, MSA, and physician specialty. The year indicator variables capture increases in spending resulting from inflation. The adjusted dollar differences therefore reflect spending in the first year of the sample, 2014

When one breaks down spending differences between hospital- and physician-owned practices by BETOS category, the 3 categories with the largest spending differentials are also the only categories that are statistically significant. Spending on imaging for patients treated by hospital-owned practices is 13.0% (*p* < 0.0001) higher than for physician-owned practices; spending on durable medical equipment is 12.9% (*p* < 0.0001) higher, and spending on unclassified services (which consists mostly of operating and recovery room claims) is 21.4% (*p* < 0.0001) higher. When spending is partitioned by site and type of care, patients treated by hospital-owned physician groups have 21.7% (*p* < 0.0001) higher outpatient facility expenditures relative to patients seen by physician-owned practices.

Patients treated by physicians in hospital-owned practices were also more likely to have paid an outpatient facility fee in a given year than patients in physician-owned practices (47.9% versus 39.1%, *p* < 0.001; see Appendix Table 4^18^). For the three BETOS categories in which we found higher adjusted spending for patients treated by hospital-owned practices, only durable medical equipment had a higher probability of positive annual spending.

The regression-adjusted spending differences in dollar values for hospital- versus physician-owned organizations closely mirror the estimated percentage differentials. For example, spending for patients treated by hospital-owned physician practices is estimated to be $280.08 more than for patients treated by physician-owned organizations, which is 6.3% higher than the unadjusted spending of $4451.80 reported for physician-owned groups in Table 3.

**Table 3:** **Claims per Member and Percent of Claimed Billed in Outpatient Setting by Physician Practice Ownership Type**

	Claims/member			Percent outpatient		
	Hosp	Phys	*p* value	Hosp	Phys	*p* value
Chemistry lab tests	4.46	5.31	< .0001	17	11	< .0001
X-rays	0.83	0.76	< .0001	24	19	< .0001
CT scans	0.23	0.22	< .0001	37	34	< .0001
MRIs	0.18	0.16	< .0001	25	18	< .0001
Ultrasound	0.19	0.19	0.006	31	22	< .0001

*CT* computed tomography, *MRI* magnetic resonance imaging

We report full regression results of the determinants of total annual spending in percentage differences and in dollar values in Appendix Table 5.^18^ Relative to the smallest physician practices (< 500 attributed patients per year), expenditures per year for larger physician organizations were not significantly different.

### Comparison of Claims per Member and Quality

Table 3 reports comparisons of claims per member and the percent of claims billed in the outpatient setting for patients in hospital- versus physician-owned practices. For four out of five tests, claims per patient were equal to or higher in hospital- versus physician-owned practices. Moreover, the percent of claims billed in the outpatient setting (versus professional setting) was greater in each case for patients treated by hospital-owned practices.

Table 4 shows no consistent difference in care quality for hospital-owned versus physician-owned practices. Diabetic patients treated by hospital-owned physician groups were less likely to receive LDL cholesterol testing (OR = 0.85, *p* < 0.0001). But women ages 50 to 64 seen by hospital-owned practices were more likely to receive breast cancer screening (OR = 1.13, *p* < .001). There was no significant difference in the remaining quality measures across ownership types.

**Table 4:** **Adjusted Differences in Quality Metrics for Hospital- Versus Physician-Owned Organizations, 2014–2016**

	Odds ratio	95% confidence interval
30-day readmission	1.04	(0.95, 1.13)
Diabetes		
Hemoglobin A1c	0.96	(0.91, 1.01)
LDL cholesterol testing	0.85	(0.82, 0.89)
Retinal examination	1.08	(0.98, 1.20)
Screening mammography	1.13	(1.11, 1.15)

The models adjusted for practice size, year, age, risk score, sex, consumer-directed health plan enrollment, MSA, specialty, and area wage index

### Sensitivity Analysis

A total of 3671 patients were excluded from the analyses, because they had spending > $100,000 in a calendar year. If these patients were included in the regression sample, the annual spending “differential for patients” treated by the hospital- versus physician-owned practices would rise from 5.8 to 6.3%.

We also examined the sensitivity of our results to alternative specifications by defining physician practice size in deciles instead of quartiles and including age and age-squared instead of categorical age variables. With these changes, the annual spending differential for patients treated by hospital- versus physician-owned practices changes from 5.8 to 6.5%.

## DISCUSSION

Similar to a previous study based on HMO claims in California, we found that a large sample of Texas PPO patients treated by hospital-owned physician organizations had higher annual spending than patients treated by independent physician organizations. Hospital-owned physician organizations had spending that was 5.8% higher, or $280 in dollar terms. Yet, hospital-owned practices were better than physician-owned groups for only one of the five quality measures examined. A recently published literature review identified multiple studies that conclude that integration of physicians into hospitals results in higher spending.^33^ This study adds to the existing literature by measuring physician-hospital integration effects with patient-level data, while simultaneously considering quality of care.

Our comparison of adjusted spending based on median prices reduced the estimated spending differential for hospital- versus physician-owned organizations by only 1 percentage point, from 5.8 to 4.9%. This result suggests that differences in utilization rather than price variation explain most of the higher observed spending for hospital-owned versus physician-owned practices. The result is consistent with the higher claims per member among hospital-owned practices for select tests reported in Table 4.

Neprash et al. conclude that the higher spending associated with vertical integration is driven almost entirely by price increases resulting from physician alignment with hospitals.^10^ However, when standardizing prices, Neprash et al. assigned the same price to services provided in both the hospital outpatient and professional setting. In contrast, we employed the BETOS classification, which tended to assign higher standardized prices to outpatient claims and lower standardized prices to professional claims. Our results are consistent with Neprash’s conjecture that higher spending associated with vertical integration results from commercial insurers following Medicare’s practice of paying more for physician services provided in hospital outpatient settings versus physician office settings, or from increased bargaining power of physician-hospital organizations.^10^

Our analysis has some important limitations. First, we only knew the network contract status of each physician in 2016. A small share of physicians likely switched between physician-owned and hospital-owned practices in the sample years 2014 and 2015, but we lacked information on these changes. When we re-estimated the regression in Table 3, restricting the sample to patients treated in 2016, the annual spending differential for patients treated by hospital- versus physician-owned practices fell from 5.8 to 5.4%. Secondly, similar to other recent studies, we lack data from prescription drug claims.^9, 10, 34^ Changes in pharmaceutical spending have been associated with changes in spending or utilization of outpatient and inpatient care in previous studies.^35, 36^ Therefore, the results in this analysis may differ if drug expenditures were included. Finally, similar to previous studies,^9–11, 34^ there may be unmeasured factors that determine vertical integration that also influence spending. Only one study attempted to use the vertical integration status of the patient’s first PCP as an instrument for integration, but the authors mention multiple weaknesses associated with this approach.^37^ Because we lack a natural experiment for measuring the effects of vertical integration, future integration of hospitals with physician practices may not yield comparable spending differences by ownership type.

## CONCLUSIONS

Data from multiple surveys suggest that hospitals are continuing to employ more physicians and forge tighter contractual relationships with doctors who prefer not to become salaried employees. Our findings, combined with previous research, suggest that increased integration leads to higher spending, with no consistent evidence of better quality. Therefore, the current debate over the health insurance coverage provisions of the Affordable Care Act has not included one of the main factors driving up healthcare spending, making coverage unaffordable.

Policy experts have offered several strategies for countering vertical integration and its consequences that require action by federal and state government agencies. Insurers can accelerate the move toward value-based payments such as accountable care organizations, in which physician groups share in financial cost savings when delivering care while maintaining quality.^38^ At the same time, the administrative and regulatory requirements for physicians should be simplified, because their complexity may be pushing physicians to join hospitals or health systems. For example, reporting requirements of quality should be reduced and standardized across health plans. Federal and state regulators should also be more vigilant in monitoring insurer contracts with health systems. Contracts should not hinder price transparency, or favor providers based on market power, versus price or quality.^39^ Attention to these policy changes is crucial for preserving a competitive, market-based healthcare system in the USA.

## Electronic Supplementary Material

ESM 1 (PDF 277 kb).
