# Supplementary material for: Annual Spending per Patient and Quality in Hospital-Owned Versus Physician-Owned Organizations: an Observational Study
Source: J Gen Intern Med. 2019 Sep 3;35(3):649–55. doi: 10.1007/s11606-019-05312-z (PMC7080686; doi:10.1007/s11606-019-05312-z)
Supplement: Supplementary file 1 — (PDF 277 kb). [file 11606_2019_5312_MOESM1_ESM.pdf]

**Appendix:** Are Hospital-Owned or Physician-Owned Organizations More Costly?

**Appendix eFigure 1: Sample Size After Each Study Exclusion**

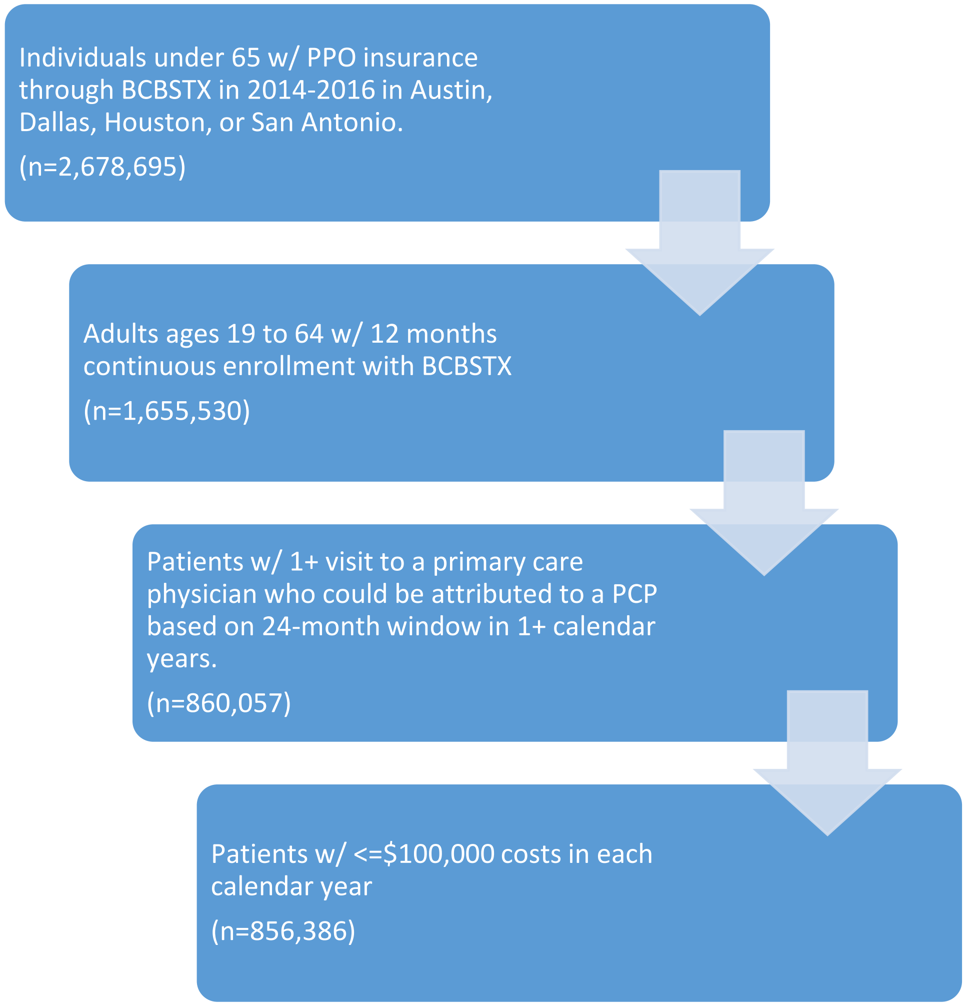

| **Appendix eTable 1: Characteristics of Patients in the Analyses Sample Compared to Excluded Patients[a]** | | |
|---|---|---|
| | Members in the Sample | Members Excluded |
| Age | 43 | 38 |
| Risk score | 1.258 | 1.061 |
| Male | 45.46% | 49.63% |
| CDHP | 22.74% | 23.60% |
| % Austin | 11.50% | 11.33% |
| % Dallas | 40.45% | 39.16% |
| % Houston | 37.14% | 37.97% |
| % San Antonio | 10.91% | 11.54% |
| Wage Index | 0.952 | 0.945 |

[a] Patients were excluded from the sample if they could not be attributed to a physician.

| **Appendix eTable 2: Distribution of Physician Specialties in Physician versus Hospital-Owned Organizations, by Year** | | |
|---|---|---|
| | **Physician-Owned** | **Hospital-Owned** |
| **2014** | | |
| Family Practice | 71.5% | 70.9% |
| Internal Medicine | 27.2% | 28.3% |
| Pediatrics | 1.0% | 0.7% |
| General Practice | 0.3% | 0.1% |
| **2015** | | |
| Family Practice | 71.1% | 72.0% |
| Internal Medicine | 27.6% | 27.1% |
| Pediatrics | 1.0% | 0.8% |
| General Practice | 0.4% | 0.1% |
| **2016** | | |
| Family Practice | 71.4% | 72.9% |
| Internal Medicine | 27.2% | 26.2% |
| Pediatrics | 0.9% | 0.8% |
| General Practice | 0.4% | 0.2% |

**Appendix eTable 3. Characteristics of Physician Organizations and Attributed Patients Participating in the BCBSTX Network by Year**

| | 2014 | | | 2015 | | | 2016 | | |
|---|---|---|---|---|---|---|---|---|---|
| | Medical Groups | | | Medical Groups | | | Medical Groups | | |
| | Physician-Owned | Local Hospital-Owned | Multihospital System-Owned | Physician-Owned | Local Hospital-Owned | Multihospital System-Owned | Physician-Owned | Local Hospital-Owned | Multihospital System-Owned |
| **Total** | | | | | | | | | |
| No. of Physician Organizations (Pos) | 1,869 | 27 | 11 | 1,869 | 27 | 11 | 1,869 | 27 | 11 |
| No. of patients | 477,841 | 63,157 | 69,462 | 477,927 | 66,127 | 77,789 | 426,827 | 61,415 | 74,318 |
| Patients per PO, mean | 256 | 2,339 | 6,315 | 256 | 2,449 | 7,072 | 228 | 2,275 | 6,756 |
| Patients per PO, minimum range | 5 | 7 | 73 | 5 | 8 | 70 | 5 | 5 | 55 |
| Patients per PO, maximum range | 57,991 | 30,806 | 38,627 | 64,168 | 29,833 | 40,349 | 58,402 | 25,411 | 39,005 |
| **Severity of patient health status (DxCG risk scores)** | | | | | | | | | |
| | 1.22 | 1.26 | 1.17 | 1.25 | 1.29 | 1.22 | 1.26 | 1.29 | 1.21 |
| **Allowed Medical Spending** | 4,163 | 4,748 | 4,374 | 4,558 | 5,078 | 4,800 | 4,656 | 5,181 | 4,818 |
| **Age** | 43.1 | 43.5 | 42.9 | 43.1 | 43.5 | 42.8 | 43.1 | 43.4 | 42.6 |

**Appendix eTable 4: Percent of Patients with Positive Annual Spending, 2014-2016 by BETOS Category and Site and Type of Care**

| | Physician-Owned | Hospital (Local + Multi) | P-value |
|---|---|---|---|
| **By BETOS category ($)** | | | |
| Evaluation and management | 99.3% | 99.2% | <.0001 |
| Procedures | 55.8% | 55.9% | 0.30 |
| Imaging | 57.0% | 56.8% | 0.15 |
| Test | 89.6% | 89.2% | <.0001 |
| Durable medical equipment | 21.2% | 22.3% | <.0001 |
| Other | 53.1% | 56.4% | <.0001 |
| Unclassified | 24.6% | 21.9% | <.0001 |
| **By site and type of care ($)** | | | |
| Professional payments | 100.0% | 100.0% | 0.13 |
| Outpatient facility | 39.1% | 47.9% | <.0001 |
| Inpatient facility | 5.1% | 5.0% | 0.01 |

**Appendix eTable 5: Determinants of Annual Expenditure Differences per Patient, 2014-2016**

| | Adjusted Percentage Difference | Adjusted Dollar Difference |
|---|---|---|
| **Hospital-owned Practice** | 5.83** | 280.08*** |
| **Organization Size, No. of patients** | | |
| **1st Quartile** | reference | reference |
| **2nd Quartile** | -0.66 | -5.68 |
| **3rd Quartile** | 0.85 | 40.6 |
| **4th Quartile** | -2.08 | -10.68 |
| **Year** | | |
| **2014** | reference | reference |
| **2015** | 6.80** | 66.92 |
| **2016** | 8.11** | 50.47 |
| **Patient Age** | | |
| **19 to 29** | 1.42 | -94.90** |
| **30 to 39** | -1.24** | -266.19*** |
| **40 to 54** | -0.2 | -151.95*** |
| **55 to 64** | reference | reference |
| **Patient Risk score** | | |
| **1st Decile** | reference | reference |
| **2nd Decile** | 50.26*** | 312.95*** |
| **3rd Decile** | 86.95*** | 643.42*** |
| **4th Decile** | 117.84*** | 1032.73*** |
| **5th Decile** | 145.3*** | 1495.02*** |
| **6th Decile** | 172.88*** | 2106.56*** |
| **7th Decile** | 204.49*** | 3046.91*** |
| **8th Decile** | 241.83*** | 4621.14*** |
| **9th Decile** | 295.19*** | 8200.65*** |
| **10th Decile** | 382.66*** | 20180.37*** |
| **Male Gender** | -2.72*** | 83.71*** |
| **Consumer-Directed Health Plan** | -0.83 | 149.00*** |
| **Index of wages and other supply costs** | | |
| **Wage Index** | -73.31 | 78839.42* |
| **Wage Index$^2$** | 63.56 | -44265.00* |
| **Cities by Year** | | |
| **San Antonio 2014** | -5.77 | -953.92** |
| **San Antonio 2015** | -5.09 | -828.19*** |
| **San Antonio 2016** | -6.33 | -872.83*** |
| **Dallas-Fort Worth 2014** | 7.44* | 109.84 |
| **Dallas-Fort Worth 2015** | 5.03* | 169.08* |
| **Dallas-Fort Worth 2016** | 3.19 | 320.46*** |
| **Austin 2014** | -0.43 | -304.56 |
| **Austin 2015** | -0.44 | -200.80 |
| **Austin 2016** | 0.74 | -65.48 |
| **Houston** | reference | reference |
| **Physician Specialty** | | |
| **Internal Medicine** | 0.42 | -50.38 |
| **Pediatrics** | 7.27*** | 155.35* |
| **General Practice** | -3.74 | 97.93 |
| **Family Practice** | reference | reference |
| **Intercept** | 616.54 | -34458.70* |

*p<0.05, **p<0.01, ***p<0.001

**Appendix eMaterial 1: Attribution of patients to a PCP**

Attribution of patients to a PCP was a multi-step process. For each enrollment month within a calendar year, we identified each patient's visits to a PCP in the same calendar year. For example, for patients continuously enrolled with BCBSTX in calendar year 2015, we first identified the most frequently visited PCP in each month from January to December of 2015. However, if an enrolled patient did not visit a PCP in 2015, we would not be able to attribute her to a PCP. Therefore, for each month in 2015, we searched for claims for visits to a PCP in a 24-month window that included the calendar year and additional months closest to that year. For example, for January 2015, we searched for the most visited PCP in each month between February 2014 and January 2016. Note than January 2015 is the midpoint of this time interval. This approach increases the probability that each patient will be attributed to a PCP, using additional information from months closest to the relevant calendar year. When processing a later month, say June 2015, we shifted the 24-month window forward to run from July 2014 through June 2016. By performing this process for each month of each calendar year, we raised the probability of identifying PCP visits, thereby raising the number of patients who could be attributed to a PCP.
